# The influence of policy narratives on evidence-informed decision-making in health policy – an analysis of Swedish healthcare policies from 1992 to 2024

**DOI:** 10.1186/s12913-026-14829-z

**Published:** 2026-06-20

**Authors:** Emma Bergstedt, Ann-Charlotte Nedlund

**Affiliations:** Division of Society and Health at the Department of Health, Medicine and Caring Sciences, Swedish National Centre for Priorities in Health, Linköping University, Linköping, 581 83 Sweden

**Keywords:** Evidence-informed decision-making, Healthcare, Governance, Health policy, Knowledge, Politics, Policy narratives, Evidence-based healthcare, Resource allocation, Priority-setting

## Abstract

**Background:**

There is a strong emphasis globally that health policy and practice can be improved by leveraging best available evidence in decision-making processes. The dynamic political context of policy means, however, that decision-making processes are highly complex. A growing field of inquiry examines how the evidence-informed approach impacts on and interacts with real-world policy development. Despite the growing recognition of the complexity of health policy development little is known about policy narratives underpinning evidence-informed decision-making. Analysis of policy narratives over time can reveal how recognition of policy problems and their corresponding solutions emerge and shift through a contestation of different ideas and interests and how they legitimise different political courses of action. The aim of this study is to explore policy narratives underpinning evidence-informed decision-making within Swedish healthcare, tracing their formulation and development from 1992 to 2024.

**Methods:**

The study is based on a textual analysis of Swedish government documents published 1992–2024. Drawing on theories of policy narratives, a thematic content analysis of 132 Swedish government-issued documents during this period was conducted.

**Results:**

Four episodes with a dominant narrative in each emerged from the analysis. They differ in ideas connected to policy problems and solutions in healthcare and evidence-informed decision-making. Besides ideas on the substance of the policy solutions, the ideas include different assumptions about problems they are solving, how healthcare actors are connected to problems and solutions, values that motivate the solutions, and assumptions about mechanisms that will promote sound decision-making and resource use in healthcare. These four policy narratives include one of efficiency, provision of information, and deliberation; one of equality, data, and standardisation; one of integration, synthesis of perspectives, and collaboration; and one of responsiveness and the local context of decisions.

**Conclusion:**

The study offers insights into the governance of evidence-informed decision-making in healthcare. The findings contribute to a broader understanding of implications of the evidence-informed approach on healthcare policy, and how healthcare policy is shaped by competing ideas, values and assumptions. They also contribute to the theoretical debate on policy narratives, and discursive practice in general in healthcare governance.

## Background

There is a strong emphasis globally that health policy and practice can be improved by leveraging best available evidence in decision-making processes, i.e. evidence-informed decision-making (EIDM). EIDM is seen to make health policies and the use of scarce resources more effective and equitable and to ensure transparency and accountability in decision-making [1–3]. Evidence-informed priority-setting processes are emphasised to ensure fair, valid, and legitimate resource allocation in health systems [4–6].

The dynamic political context of policy means, however, that decision-making processes are highly complex [7–9]. To improve policy and practice it is important to not only understand how decisions emerge within real-world political environments [10–13], but also to explore how the evidence-informed approach impacts on, relates to and interacts with real-world policy development [14–16]. Examples of recent studies on how the evidence-informed approach interacts with complex political environments include how actors use the approach to promote their interests [17], the politics of the emergence of government-funded knowledge brokering organisations [18], and how regulatory agencies established to depoliticise policy become a source of subsequent politicisation [19].

Translating evidence into policy and practice is a major area of health research that focuses on how to bridge the gap between research and action [8, 20, 21]. Studies on knowledge translation often investigates how evidence is communicated, understood, and applied by policymakers and practitioners, as well as barriers that can prevent its use [10, 20, 22]. The role of narrative and story-telling as a tool for researchers to influence decision-making has been given some attention. These studies conclude that those seeking to ensure uptake of evidence in policy can communicate more effectively if they narrate complex topics [23, 24].

Narratives are however not only tools for persuasion, they are the language of human action [25, 26]. Narratives tie together separate events or experiences into a meaningful and coherent whole so that complex issues can be clarified, and ambiguity reduced [27, 28]. A central function of narratives is that they produce order and meaning and present reality in such a way that it becomes more manageable [28]. In policy, actors can connect events, actors, and purpose by negotiating and handling different storylines about policy problems and solutions [26]. The recognition and legitimisation of policy problems and their corresponding solutions emerge through a contestation of ideas and interests [29]. Analysing policy narratives over time is useful for disentangling how different ideas about policy issues are highlighted and legitimise different political courses of action [30, 31].

Despite the growing recognition of the complexity of health policy development and implications of the evidence-informed approach on policy, little is known about policy narratives underpinning evidence-informed decision-making. The aim of this study is to explore policy narratives underpinning evidence-informed decision-making within Swedish healthcare, tracing their formulation and development from 1992 to 2024. The study is based on a textual analysis of Swedish government documents. The paper offers insights into the governance of evidence-informed decision-making in healthcare. It contributes to a broader understanding of implications of the evidence-informed approach on healthcare policy, and how healthcare policy is shaped by competing ideas, values and assumptions. It also contributes to the theoretical debate on policy narratives, and discursive practice in general in healthcare governance.

### Understanding narratives as informing policy in dynamic political settings

In this study, policy is taken to mean how governments respond to societal problems by creating solutions that aim to affect behaviour in the actors that are targeted by the policy and ultimately reach societal goals (see e.g. [32]). When public policy is developed, decision-makers need to connect events, action, and purpose by establishing what the problem is and what is causing it, what the solution is and who is going to provide it, and why. The policy process is deeply intertwined with the construction of political problems and their immanent solutions [29, 33]. Policy domains, target populations, goals, and viable solutions do not simply exist as an objective reality; they emerge only once an issue is recognised as a political concern. A problem is often shaped prior to the articulation of goals and can even emerge in response to a proposed solution. Both the objectives and the strategies to achieve them are open to negotiation and reinterpretation [29, 34, 35].

A policy narrative serves as a central mechanism in guiding political action [25, 28]. Narratives rely on interpretations of causality, dynamics, and epistemic claims. What qualifies as a problem is contingent upon the narrative through which it is discussed [36]. These narratives frequently include “causal stories” that establish a coherent and persuasive account of the problem’s origins, development, and resolution [37]. Through this mechanism, narratives provide justification for specific actions and serve to legitimize particular political agendas [38, 39]. Moreover, the way a problem is narrated often draws upon precedents or existing solutions to similar issues [33]. In doing so, policy narratives reinforce and stabilize underlying assumptions within the policy process [38].

In this study, the analysis of policy narratives is understood as an interpretative approach to analyse policy. The analytic focus in interpretive policy analysis is meaning-making and sense-making in policy development [40]. Studying policy narratives over time reveals how policies evolve through a sequence of ambiguous and often conflicting claims, reflecting inconsistent responses to diverse ideas and interests [26]. Narratives are reinterpreted in dynamic political settings; they evolve, adapt, and compete for dominance [41]. A dominant narrative can contribute to policy stability. A dominant narrative will, however, always be contested by counter-narratives that can influence or disrupt the dominant narrative. Counter-narratives challenge or resist the dominant narrative by bringing attention to overlooked issues, contesting power relations and values, and advocating for alternative approaches [27]. A counter-narrative can become a dominant narrative, and different policy narratives the give meaning to the same issues can exist in parallel.

Policy documents and their inherent policy narratives [38] are powerful governing tools, that hold interplay of different rationales that each simplify the phenomenon they are seeking to govern. The documents send signals to both other governing actors and the public on how issues of concern should be handled and how matters are valued [31, 42]. Analysing narratives in policy documents is a useful theoretical perspective for both unveiling the temporality and contextuality of policy, and for disentangling shifts in values and power relations in governance [30]; e.g. how issues are depoliticised and re-politised through shifts in or beyond the boundaries of conventional visible politics. These shifts are often related to the temporal character of political problems and politics of time.

According to our view, politics of time refer to how governance and institutions evolve over time, and how policies are sequenced (formulated, introduced what and where, how they are implemented, practiced and become institutionalised, and what outcome and consequences they might have). The concept is used here to refer to the temporal character of politics and the shifting modes of operation that result from the shifting policy narratives. Hence, by studying policy narratives we can shed light on the power of narratives that in the end govern healthcare and its associated politics.

In this study, a policy narrative is operationalised as a coherent storyline that includes ideas about policy problems and solutions. These include ideas about the substance of the policy solutions and the problems they are perceived as solving, how different actors are connected to problems and solutions, values that guide and motivate solutions (e.g., ideas about importance and valued ends), and assumptions about causality (e.g., ideas about the causes of problems or what will affect behaviour in the desired direction).

### The Swedish healthcare system

Sweden’s healthcare system operates across three governance levels: national, regional, and local/municipal. Health care is predominantly funded by public means and politically governed by direct-elected politicians on the three levels. The main responsibility for financing and providing healthcare is with regional authorities (formerly named county councils) and local authorities. Most of healthcare services are provided by regional authorities. The government directs healthcare occurring under regional and local authorities through legislation and regulation, guidelines and evaluations produced by government agencies, and government grants for specific activities [43].

In Sweden, the evidence-informed approach to decision-making and policy in healthcare is prominent. There are many governmental agencies tasked with providing decision-makers in healthcare with research evidence and information. There are governing bodies on all levels of the healthcare system tasked with handling research advice. In the case of priority-setting pertaining to resources on all levels of the healthcare system, three ethical principles for priority-setting were stipulated by law in 1997. These are reflected in National Guidelines issued by the National Board of Health and Welfare (NBHW), which in their evidence-based guidelines not only recommend appropriate medical treatments but also provide recommendations to policymakers on how healthcare resources should be prioritised [44, 45].

## Methods

The study is an analysis of texts in Swedish government documents. The selection of documents is part of a larger dataset. This dataset includes documents published from 1982 to 2024. The starting point 1982 followed the adoption of the Health and Medical Services Act, which marked a significant legislative change in Swedish healthcare.

The structures and instruments to facilitate evidence-informed practice and decision-making in healthcare is commonly called *kunskapsstyrning* in Swedish which can be translated to “knowledge governance.” Swedish knowledge governance is often referred to as a form of soft governance comprised of guidelines and advice for healthcare decision-makers. There is, however, no common definition as to what type of governing mechanisms this governance entails [46]. In this study, selection of policy documents has been on the notion of “knowledge governance” in Swedish healthcare. The first time the term was identified in a government document was in a report from 1993. Hence, the analysis was decided to start 1992 with the government instructions for the committee that produced said report.

In total 132 documents spanning from 1992 to 2024 are included in the deep analysis (a list is available upon request). These are a) two types of documents that outline government funding for healthcare: budget bills for healthcare (one per year 1993–2024) and funding agreements with the regional and local authorities regarding healthcare activities (one per year 1993–2024, except for 2005 and 2008 which are missing from the government archives). These agreements are negotiated through the Swedish Association of Local Authorities and Regions (SALAR), an interest organisation for local government in Sweden with all regions and municipalities as its members; b) Official Reports of the Swedish Government. Government-commissioned investigations into specific issues are a cornerstone of Swedish policymaking. These expert reports are typically led by independent experts, academics and senior civil servants. All major commissioned expert reports on healthcare governance during this period were included. 39 in total (this was determined by collecting reports by delegations that are referenced in documents); c) government instructions for the expert committees tasked with producing the reports (28 documents); and d) three other documents on national knowledge governance published in this period (a regulation, a memorandum, and a declaration of intent).

The documents were collected and read by the first author. Emerging patterns and variations in the texts were categorised by both authors in several meetings during which the analysis and findings were discussed. The texts were abductively analysed [47] using a framework approach to thematic content analysis [48]. Drawing on the theory of policy narratives guiding political action by constructing policy problems and solutions, and that policy shifts over time involve dominant narratives, parallel narratives, and counter-narratives, several themes emerged. In order to discern narratives, passages illustrating the following were extracted from documents: What is written about “knowledge governance” or “knowledge-based care”? (e.g., definition or something general/summarizing); Which instruments, bodies, and processes are referred to as knowledge governance/governing with knowledge?; Who is supposed to provide the solutions, and who is expected to use them? What problems do they solve?; How and for whom?; What are the higher goals being pursued? Are there alternative solutions or counterarguments against existing solutions?

These key areas were then summarized chronologically. While the same ideas may appear across the whole period, a pattern emerged where ideas and arguments were more distinctly articulated within specific episodes, forming a dominant narrative within a distinct timeframe. Specific attention was given to whose knowledge the assumptions in different solutions highlight. Four episodes with a dominant narrative in each emerged in the analysis. The patterns were summarised into main ideas about policy problems, solutions, and main bearers of knowledge in each episode.

During the analysis, the complexity of “knowledge governance” became evident, involving several actors and types of solutions connected to various constructed problems. These also shifted over time. In the presented result section, a new episode starts when there is a significant shift of a new dominant policy narrative, i.e. when there is a significant shift in how either problem or solution is expressed. The narratives from one episode continue into the others, but only the dominant narrative in each episode, and how it eventually started shifting, are described in the findings. The narratives are kept brief; they do not claim to reflect or reconstruct episodes and narratives fully. However, they shed light on major policy narratives related to the temporal construction of “knowledge governance”. The findings have been presented to a group of senior researchers with expertise in Swedish healthcare, to ensure the reliability.

## Results

In this section four episodes spanning from 1992 to 2024 are presented. Each episode has a dominant narrative which is summarised into main ideas about problems and solutions, and the main bearers of knowledge (Table 1). Besides ideas on the substance of the policy solutions, the ideas include assumptions about problems they are solving, how healthcare actors are connected to problems and solutions, values that motivate the solutions, and assumptions about mechanisms that will promote sound decision-making and resource use in healthcare. The emergence of a new episode does not necessarily imply the end of narratives from preceding episodes; rather, it often marks a shift in their prominence, as they become less dominant but continue to inform the present.

**Table 1:** Summary of policy narratives on national knowledge governance in healthcare in Sweden during the years 1992–2024

Summary of policy narratives on Swedish national knowledge governance in healthcare				
Episode Key area	1992–2003	2004–2012	2013–2018	2019–2024
Essence of dominant narrative	Information given to decision-makers and the deliberation between them will develop decision-makers’ knowledge and enable efficient and effective resource use in healthcare	Indicator-based quality monitoring and integration of data production and management will provide decision-makers with knowledge and enable nation-wide equality, equity, and efficiency	Collaboration, synthesis, and coordination will produce and apply best available knowledge as well as enable integrated and efficient governance and healthcare	Person-centeredness and emphasis on the local context for health professionals will enable responsive, efficient, and knowledge-based healthcare
Main ideas about problems	There is an increasing mismatch between healthcare needs and available resources in healthcare.	There are regional disparities in health and healthcare provision. There are insufficient tools to monitor healthcare performance and insufficient research data on which to base decisions.	There are long waiting lists in healthcare and healthcare delivery is fragmented. Regional and local authorities are still unable to implement national directives.	Healthcare organisations lack capacity and are insufficiently responsive. Healthcare practice involves unnecessary care.
	Healthcare practice is not sufficiently focused on patients.	National knowledge governance is not sufficiently active and systematic.	Measuring and administration have become increasingly burdensome for health professionals.	National knowledge governance involves excessive bureaucracy, a disproportionate number of guidelines, and management by too many actors.
	There is a lack of research, evaluations, and assessments that can improve healthcare practice, determine cost-effectiveness, and guide priority-setting in regional authorities.	Regional and local authorities are unable to apply the national guidelines and to explicitly prioritise resources.	Contradicting guidelines by national agencies as well as other actors causes confusion for decision-makers	There is insufficient evidence about the results of national knowledge governance.
Main ideas about solutions	Reserch reviews, guidelines, and evaluations of cost-effectiveness can provide a basis for agreement with patients and between decision-makers on resource allocation.	Measuring different aspects of healthcare delivery will enable monitoring and evaluation.	Increased collaboration between different health-related actors will coordinate and integrate the production of directives as well as their implementation.	Increased involvement of patients and significant others in decisions will improve health and enable better use of healthcare resources.
	Explicit priority-setting in clinical decisions and healthcare budgets will reveal options and trade-offs in resource use.	Indicators will enable standardisation and comparisons between different parts of Sweden. Open comparisons will motivate healthcare providers to improve healthcare and resource use.	Increased collaboration will also reveal different stakeholders’ perspectives and enable the synthesis of them into uniform recommendations and the provision of best available knowledge.	Health professionals need support in not choosing treatment options that do not produce value for patients.
	Information from national agencies and regional/local civil servants, together with training and deliberation, will develop decision-makers’ knowledge, which will enable them to use and distribute resources efficiently and effectively.	Centralisation of care will enable systems that can handle the uptake of research, produce research, and allow the integration of data in management.	Collaboration and standardisation in healthcare delivery will integrate healthcare and enhance implementation of national knowledge governance directives.	Competency maintenance and improved work environment for health professionals will increase accessibility in healthcare, as well as health professionals’ practical conditions to develop their knowledge.
		Data and numbers will provide decision-makers with knowledge and enable equal, equitable, and efficient care throughout the country.	Collaboration and coordination will make knowledge governance more efficient and effective, and make healthcare timely, equal and knowledge-based.	Emphasis on the active participation of patients and on the local context for health professionals will enable responsive, efficient, and knowledge-based healthcare.
Main bearer of knowledge	Healthcare professionals and local/regional politicians, as the main decision-makers in healthcare	Actors producing numbers and data; i.e., researchers and civil servants	Expert groups synthesising different perspectives into uniform recommendations	All actors’ knowledge is potentially involved, since emphasis is on the integration of the views of patients and significant others, and on the local environment for health professionals

### 1992–2003: Information and deliberation will develop decision-makers’ knowledge and enable efficient and effective resource use in healthcare

At the beginning of the 1990s, the notion of knowledge governance started to emerge in Swedish national health governance. In 1992, the government commissioned a committee of inquiry to analyse and assess resource needs of the healthcare system, and discuss how healthcare should be financed and organised at the overall societal level [49]. One of the main tasks assigned was to consider different cost-saving opportunities. The background given was rising resource demands due to an aging population, unmet healthcare needs, and medical and technological advancements, which could both reduce costs through improved treatments and increase costs by enabling new, expensive, interventions [49]. In a report authored in 1993 by an expert group associated with this committee, knowledge governance was described as a “method related to professional governance” [50, p. 326] which is used to assess costs and effects of medical technology. According to the report, the method involves “development of treatment recommendations using knowledge reviews, known as synthesis studies” and that it”is probably best suited when questioning proven experience or reconsidering old routines with the help of scientific results” [50, p. 326].

Initially, knowledge governance mainly referred to research reviews and analyses of cost-effectiveness provided by national authorities, particularly The Swedish Council on Technology Assessment in Healthcare (SBU), established in 1987. As described in a government budget proposal, the main assignment of SBU was to “contribute to the rational use of resources within healthcare so that healthcare providers can deliver the best and most comprehensive healthcare possible within the given financial framework with regard to current scientific knowledge and proven practices” [51, p. 201]. It states that” medical methodology is an activity with significant development potential and should be regarded as a strategic policy instrument for Swedish healthcare” [51, p. 203].

Another government agency that came to play an important role was The National Board of Health and Welfare (NBHW) that in 1996, was commissioned to develop clinical guidelines with recommendations for diagnosis and treatments for different patient groups [52]. In a funding agreement between the government and local and regional authorities, the guidelines are described as enhancing the patients’ opportunities to receive knowledge-based care by providing a basis for individual agreements with the patients [52]. More information given to patients and their significant others was stated as a prerequisite for increased engagement by them in decisions. The agreement also pointed out that “the greatest rationalisation gains are likely to be achieved by health professionals themselves discussing and agreeing on the ‘right’ interventions for different conditions, with the help of research findings.” [52, p. 6]

In 1997, parliament adopted a law saying that the basis of resource allocation on all levels of the healthcare system should be explicit priorities guided by certain ethical principles [53]. Rising healthcare demands and the worsening financial situation in Sweden were explained as intensifying the need for explicit priorities [53, 54]. In the government investigation that preceded the parliament’s decision, guidelines for priority-setting in regard to healthcare resources were suggested. NBHW was commissioned to develop methods to operationalise the application of these guidelines [55]. The aim was described in a funding agreement to ensure that all national guidelines were gradually supplemented with advice that can serve as a foundation for decisions in priority-setting [55], ensuring the “fulfilment of the long-term goal of integrating the issue of priorities into the planning work of healthcare providers at all levels, resulting in conscious and open priorities” [56, p. 7].

This development highlighted the role of local and regional politicians in explicit priority-setting of healthcare resources, since they were responsible for the overall allocation of resources among local and regional authorities. In a report written by a committee investigating the application of the guidelines for priority setting, the role and knowledge development of local and regional politicians in priority-setting were emphasised [57]. Priority-setting was declared as requiring extensive dialogues between politicians and different groups and the public, and politicians required good factual material and information to be able to make sound decisions, as emphasised in the report; “Cost-effectiveness analyses and evaluations are tasks that have so far been relatively undeveloped in healthcare. (…) Political representatives must become better at requesting and commissioning such well-prepared factual and analytical documentation from the administration that is adapted and relevant to the priority setting process and that provides a basis for making the decisions that are necessary in this context” [57, p. 97].

In the final report by the committee investigating resource needs and organisation in the healthcare system [58], knowledge governance is emphasised, although the term ‘information governance’ is preferred since “information in itself is not knowledge. Knowledge does not exist unless someone has acquired it.” [58, p. 119]. The report suggests that a strategy and coordination of this type of national governance should be developed. The emergence of knowledge governance is described as the following: “information, education, and all forms of knowledge development are gaining increasing importance as a means of influencing and governing healthcare. Through its governance with information of various kinds, the state wants to provide opportunities for knowledge development, and ultimately a change in behaviour, in healthcare issues” [58, p. 131].

In summary, the initial overall narrative on knowledge governance mainly referred to solutions meant to solve the problems of the increasing mismatch between healthcare needs and available resources in healthcare. The main assumptions in this narrative were that research information to decision-makers and explicit priority setting would facilitate efficient and effective resource use. Explicit priority-setting in clinical decisions and healthcare budgets would reveal options and trade-offs in resource use. The provision of research information and cost-effectiveness evaluations to decision-makers (in this case the main decision-makers are healthcare professionals and local/regional politicians) would facilitate deliberation and agreement and develop their knowledge. This would in turn make them use resources efficiently and effectively.

During this episode, a parallel narrative stressing the need for efficient and equivalent use of healthcare resources emerged. The growing number of studies and assessments of health and healthcare made regional disparities more apparent. There were changes in the healthcare system, with more private actors, and new forms of management during this episode. This was described as posing challenges which required evaluations and monitoring of performance in healthcare organisations [59]. The patients should also have access to information due to the advances in information technology which enabled systems for more information to patients [60].

### 2004–2012: Indicator-based quality monitoring and integration of data production and management will provide decision-makers with knowledge and enable nation-wide equality, equity, and efficiency

As of 2004, a new phase emerged with narratives that increasingly articulated equality and equity in healthcare. In 2004, the National Guidelines commissioned by NBHW also included advice about how to allocate and prioritise resources, aimed at decision-makers responsible for planning healthcare in the regional authorities, to support that “healthcare resources are used efficiently, allocated according to need, and governed by systematic and transparent priorities” [61, p. 6]. It was also argued that national guidelines should include quality indicators which reflected the increasing focus on systemic monitoring [62]. Management structures in regional authorities to apply the guidelines issuing from national authorities were also funded by the government [63].

Measuring, monitoring, and comparing performance in healthcare was central in this episode. A prominent example of this was reports on “open comparisons.” Open comparisons were part of a new “national strategy for good healthcare [which] expresses a more active and systematic knowledge governance of healthcare” as expressed in a funding agreement [63, p. 3]. It was an initiative funded by the government to measure and compare quality, results, and efficiency between different regions and healthcare providers [62]. The aim was to provide decision-makers and the public with numbers based on uniform national indicators. The assumption was that “when data is used in open comparisons, the pressure to improve data quality and develop new data sources increases” [62, p. 13]. The aim of the strategy of open comparisons, as expressed in a budget proposal, was to “through increased transparency, stimulate the development of equal, safe and efficient healthcare of good quality. The vision for the strategy is: up-to-date knowledge when you want it and how you want it” [64, p. 50].

An important source of information in the reports on open comparisons was national quality registries. The registries contained individual-level data on diagnoses, interventions, and outcomes. They were cited as enabling “major savings” both financially and in terms of reduced suffering for patients [62]. The potential of the use of the registries when it came to improving healthcare and data for research were, however, described as insufficient. The government made substantial investments in the development of quality registries to, among other things, integrate it with other IT systems in healthcare, and to create “better access to and use of quality registers for research and innovation purposes, increased transparency, and access to data also for patients and ultimately better and more equitable care” [64, p. 69].

In line with a more active and systematic knowledge governance was centralisation of healthcare provision in this episode. In 2010, the establishment of regional centres for cancer treatment began with funding support from the government. This was stated as part of “efforts to improve knowledge governance regionally and nationally” [65, p. 28] and that it “in a concrete way will strengthen knowledge formation and dissemination within cancer care. The aim is to increase the quality of care and improve care outcomes, as well as to make the use of healthcare resources more efficient” [66, p. 54]. Another aim was stated as “achiev[ing] more equal cancer care, increase[ing] the quality of care and offer[ing] faster care” [64, p. 85].

Information on the background of this decision can be found in government reports. In 2007, a government report investigating the potential for changes in task allocation and structure to meet the challenges that public sector organisations will face in the future, suggested the establishment of “regional knowledge centres” [67]. The main assumption was that national knowledge governance of healthcare would and should increase, since knowledge governance was a tool to achieve both equity and equality in healthcare. Regional authorities, however, needed systems to implement these directives. The centres would be tasked with collaborating with national-level organisations through knowledge dissemination and knowledge development, and the implementation of knowledge into practice within healthcare organisations [67]. In the report preceding the decision to establish regional centres for cancer treatment, this was repeated, and it was suggested that “within the framework of the proposal for a unified cancer service, we believe that there is a clear need for strengthening knowledge development and knowledge governance in the field of cancer, a resource that can receive and evaluate new findings and provide a basis for decisions on the introduction, or withdrawal, of medical methods (a regional HTA resource). Education, clinical cancer research, implementation of new findings, and follow-up and evaluation in cancer care should thus be functionally integrated” [68, p. 234].

In summary, during this episode, there was an overall call in government documents for knowledge governance to become more strategic, active, and systematic. The main narrative on knowledge governance referred to solutions meant to solve regional disparities in provision and quality of healthcare. The assumption was that equality, equity, and efficiency could be achieved by monitoring performance in healthcare and by centralising healthcare provision. The ideas included measuring different aspects of healthcare performance which would enable monitoring and evaluation and provide decision-makers with knowledge. Quantitative data and indicators would enable standardisation, which would enable comparisons between different parts of Sweden, and open comparisons would motivate healthcare providers to improve healthcare and use resources efficiently. Centralising care would enable systems that could handle the uptake of research, produce research, and enable the integration of data in management.

However, a parallel narrative, that would eventually become more dominant, stressed that the systems, databases, and registries required extensive administration and reporting. The administrative load on healthcare professionals and the use of limited data as a basis for making decisions in complex settings, were increasingly viewed as causing problems [69]. The number of actors involved in knowledge governance also increased, and advice produced by different actors, governing bodies, and instruments were perceived by decision-makers as contradictory at times. Moreover, the lack of implementation of national directives remained a problem [70].

### 2013–2018: Collaboration, synthesis, and coordination will produce and apply best available knowledge as well as enable integrated and efficient governance and healthcare

In 2013, the government commissioned an investigation into how healthcare services can use professionals’ resources in more appropriate and efficient ways. The instructions to the committee says that “the requirements for reporting to registers and other monitoring systems are considered by the professions to be far too burdensome and are not always perceived as relevant. (…) The extent of the administration contributes to negative perceptions of the work environment and to a decrease in trust in the governance and management of healthcare among its staff” [71, p. 4].

In the subsequent report, it is stated that the increasing and varied forms of governing healthcare, where knowledge governance is one of them, is causing administration and ambiguity for health professionals, and that increased collaboration between actors in knowledge governance is needed; “an example of the consequences of multiple governance is the ‘knowledge governance’ carried out by many different actors (…) Despite extensive work by many actors with a good purpose, there are obvious risks that the sum of the work is a multitude of “governance signals” that primarily create ambiguity for the professions. The risk of overlaps and duplication of work is also obvious” [72, p.533].

A prominent example of an attempt to coordinate and synthesise national knowledge governance was the establishment in 2015 of the national “Council for Governing with Knowledge” [73]. The council consisted of representatives from different national authorities and an advisory group of political representatives from regional and local authorities that was tasked with agreeing on what they commonly need from national authorities in terms of knowledge. The aim of the council was stated as efficient use of resources in knowledge governance and increasing its impact among regional and local authorities. A central task of the council was also to work to ensure that the views and experiences of patients and service users are taken into account in the agencies’ knowledge governance [73].

The memorandum preceding the council’s establishment stated that collaboration and coordination between authorities, sectors, and governing levels when it came to knowledge governance would solve many problems—e.g., duplication of work, unclear responsibilities, contradicting advice, and lack of implementation of national directives [46]. It could also facilitate the integration of perspectives of patients and service users in national directives, as well as facilitate the synthesis of health professionals’ judgements in areas where research evidence was deemed insufficient. The assumption was that a council with representatives from different entities would enable agreement, which would promote improved evidence-based practice in both healthcare and social services, and reduce costs, since governmental authorities would have “the opportunity to decide, based on a broader knowledge base, which needs regarding knowledge governance should be prioritised” [46, p. 94].

Collaboration and coordination were also emphasised as important in healthcare delivery. In 2015, standardised care pathways were introduced in cancer care. The main problems they were described as solving were long waiting lists for cancer patients and regional disparities in care [73]. Standardised care pathways were nationally defined processes that aimed to coordinate care across services to facilitate timely, integrated, equal, and knowledge-based care for cancer patients throughout the country [74]. In 2018, regional actors established an extensive nation-wide system for knowledge governance in healthcare, comprised of expert groups in various areas. In an agreement with SALAR, the government commissioned the system to develop guidelines for standardised care pathways in more service areas to expand the solution to more patients. Patient contracts were instructed to be a central part of the guidelines for care pathways [74].

In summary, the overall narrative on national knowledge governance during this episode mainly referred to solutions intended to solve the problems related to a lack of implementation of national knowledge governance; delayed and fragmented healthcare delivery, and problems that the increasing number of actors and directives in knowledge governance were perceived as causing in the healthcare organisations. The assumption was that increased collaboration and synthesis of perspectives would integrate and improve knowledge governance and make it more efficient. The notion of ‘best available knowledge’ became more pronounced with the emphasis on collaboration and synthesis of perspectives. Moreover, the assumption was also that coordination and standardisation in healthcare delivery would integrate healthcare, make it more equal and timelier, and enhance implementation of national knowledge governance directives.

However, the emphasis on patient contracts in care pathways hinted at a solution to another problem which would eventually form the dominant narrative on knowledge governance. The aim of patient contracts was stated as contributing to more responsive healthcare and to involving patients and their significant others more in care and treatment [75]. Making healthcare more responsive was part of a major government drive on shifting Sweden’s focus to primary care, which was the start up for an episode with at new dominant narrative.

### 2019–2024: Person-centeredness and emphasis on the local context for health professionals will enable responsive, efficient, and knowledge-based healthcare

In 2019, the new knowledge governance system started issuing guidelines on care pathways which are now called “personcentred and coordinated” instead of standardised in the funding agreement with SALAR [76]. A subsequent agreement states that “the government’s investment in person-centred and coordinated care pathways is part of knowledge governance in healthcare” [77, p. 19 (33)] and stressed that they do not create unnecessary administration for health professionals. Care pathways were described as “bringing care closer” to patients, which was stated as a “prerequisite for long-term sustainable healthcare where the transition to local care is central” [77, p. 19 (33)].

In a budget proposal, person-centeredness and patient contracts were described as “areas that are central to the work of transitioning to local care with primary care as the hub” [78, p. 42] and that although the consequences of the COVID-19 pandemic became the government’s main focus in the beginning of this episode, the work of shifting focus to primary care continued. The assumption was that “developed local care with strengthened primary care can provide improved conditions for socio-economically efficient healthcare where available resources are used in the best way. The development towards better and closer care is considered to increase the conditions for patients to be more involved in their own care, which in turn means increased compliance with treatment advice, which contributes to better health and more efficient healthcare” [79, p. 40].

In a government report investigating “a national coherent system for knowledge-based healthcare”, a concern for “overuse” by the government of the regional authorities’ knowledge governance system was expressed by regional actors, since the regional authorities were perceived to lack the resources needed to implement a large number of care pathways. In the report, a coherent system between actors at national, regional, and local levels was assumed to increase collaboration and follow-up, but a transparent and systematic process for arriving at what areas to prioritise was also declared as necessary [80]. In a declaration of intent on a “joint direction for coherent and effective knowledge governance in healthcare” between governmental and regional actors, the problem of the excessive number of actors and directives in knowledge governance was emphasised: “This creates ambiguity in how different knowledge supports relate to each other and which ones should be guiding. It also risks making it more difficult for decision-makers and health professionals to use knowledge support and could ultimately lead to unequal healthcare” [81, p. 1 (3)]. An “integrated chain” with clear responsibilities among all actors was stated as necessary, as was a shift in focus from the production to the implementation of directives and the follow-up of initiatives. In the same vein, de-implementation of low-value care by health professionals was stated as central for knowledge-based healthcare [81].

During this episode, the work environment for health professionals and competency maintenance was stressed in government documents. The challenges facing healthcare were emphasised: “Swedish healthcare is characterised by long waiting lists, poor accessibility, and an insufficient number of hospital beds. (…) In order to increase accessibility, it is important that more people want to work in healthcare. To achieve this, the work environment and working conditions need to be improved and the supply of skills needs to be secured” [78, p. 45]. A prominent example of funding in this context was the establishment of a council for competency maintenance in healthcare in 2020 [79]. These factors were also described as important in the context of health professionals’ knowledge development and knowledge-based healthcare. Health professionals were considered as needing more time and practical conditions in their workplace to learn and keep up-to-date about scientific discoveries, and for dialogue with colleagues and other actors. These practical conditions were declared as the responsibility of local management to develop, but could also be supported by national authorities, such as the new council for competency maintenance [80].

In summary, the main narrative on knowledge governance in this episode refers to solutions to the problems of inadequate responsiveness in healthcare provision. The assumption was that person-centeredness and ‘closer care’ would enable better use of healthcare resources. More emphasis on work environment and competency maintenance was assumed to increase accessibility in healthcare as well as health professionals’ practical conditions to develop their knowledge. The problem of the excessive numbers of actors and directives in knowledge governance remained. De-implementation of ‘low-value care’ came into focus in this context.

However, the persistent problem of the excessive numbers of actors and directives in knowledge governance, as well as lack of implementation and follow-up of knowledge governance initiatives, promoted ideas about a national coherent system, or an ‘integrated chain’ of actors involved in knowledge governance on different levels, with clearer roles, as well as a process of explicit priority-setting when national initiatives are developed. A more transparent prioritisation of knowledge governance initiatives, a clearer division of responsibility, and follow-up of knowledge governance initiatives were assumed to improve resource use. These ideas about problems and solutions hint at possible emerging narrative and future political course of action.

## Discussion

This study shows that the aspirations of knowledge governance, the Swedish version of facilitating evidence-informed decision-making in healthcare, have been many during the last three decades in Swedish national healthcare policy. The structures and instruments that have been established to either “govern knowledge” or “govern with knowledge” aim to make healthcare efficient, effective, equitable, equal, integrated, timely and responsive. They also aim to make decision-making and resource allocation more rational and transparent. Several of these promises of evidence-informed decision-making are prominent globally in the health field [1–3, 5, 6]. These aspirations are of course important, and so is finding the adequate role for evidence in decision-making and priority-setting of resources.

The dynamic political context of policy means, however, that decision-making processes are highly complex. Previous research on the role of evidence in informing policy and practice stresses that, to improve policy and practice, it is important to understand how decisions emerge within real-world political environments [7, 9, 11, 22]. Moreover, a growing field of research explores how the evidence-informed approach impacts on, relates to and interacts with these environments [14–19]. In other words, to reach the goals that evidence-informed decision-making is striving for, it is important to acquire insight into the inevitable politics and dynamics of policy development.

This study contributes to this field by exploring policy narratives underpinning evidence-informed decision-making within Swedish healthcare over time. The analysis of policy narratives can reveal how recognition and legitimisation of policy problems and their corresponding solutions emerge and shift through a contestation of ideas and interests [26, 29, 33]. Analysing policy narratives over time is useful for disentangling how different ideas about policy issues are emphasised and legitimise different political courses of action. [25, 28, 30, 31]

The findings in this study highlight political action regarding evidence-informed decision-making in Swedish healthcare from 1992 to 2024 by revealing shifting ideas linked to policy problems and solutions. They uncover a pattern in the development of the evidence-informed approach in Swedish healthcare, which may also be observed in other national contexts and other sectors. The evidence-informed approach can be seen as a means of depoliticising policy, helping to make resource allocation less contentious [13, 19]. These findings demonstrate that the approach is not static but shifts in response to the politics of the time. This study may provide a basis for comparing variations across countries and contexts, illustrating how the notion of evidence-informed decision-making influences policy and interacts with other elements in the policy environment over time.

The analysis of policy narratives over time reveals the dynamics of policy, i.e. the non-linearity of policy development [30, 31, 33]. A problem is often shaped prior to the articulation of goals and can even emerge in response to a proposed solution. When solutions are introduced, they will often shape another problem. The same values can be ubiquitously prevalent, but whether they come to the fore, and how, depends on the formulation and legitimation of problems and solutions. Some examples of this in this study are that the solutions in the first episode of increasing studies and evaluations can be said to have amplified another problem – regional disparities became more obvious. The advances in information technology can be said to have shaped the problem of lack of information for patients. The idea of phasing out treatments was evident already in the first episode but has come back in another form in the last one. The idea of engaging patients more in healthcare decisions is prevalent in all four episodes, but in four different ways. Currently, national knowledge governance appears to be viewed as both a problem and a solution in Swedish healthcare. The solutions to reach efficient, equal and equitable healthcare have accumulated and are now formulated as risking unequal and inefficient healthcare.

The connection between problems and solutions affects power relations in healthcare since solutions always serve different interests. In this study, the main instruments in Swedish knowledge governance over the years are highlighted. It reveals that knowledge governance involves many forms of governing mechanisms. These instruments have had significant implications for healthcare systems, both indirectly through the ideas they embody and directly when they are accompanied by targeted funding that drives organisational change. Moreover, different mechanisms involve different forms of evidence, highlight different actors’ knowledge, and focus on different patient groups. This is central when it comes to influence of different stakeholders in healthcare, and what areas are prioritised in terms of resources.

The analysis of policy narratives further unveils the contextuality of policy and how the language of expertise is reflected in politics. The policy must follow the logic in the context that it attempts to govern. In the context of healthcare, experts and researchers play very prominent roles, and policy must reflect the rationale used by experts to gain legitimacy [42]. The healthcare context is traditionally permeated by a scientific and biomedical rationale. In this rationale, knowledge development and efficient use of resources are mainly promoted by data, evaluations, and prospective studies that measure effectiveness. There is, however, a competing rationale contradicting the evidence-based approach, where knowledge development and the effective use of resources in healthcare are mainly promoted by different dialogical activities, such as co-creation and co-ideation, rather than data and expert guidelines [82]. Compromises of these different rationales can be viewed in the temporal construction of Swedish knowledge governance, particularly in the last decade.

Ideas however do not have boundaries neither when it comes to countries nor academic fields. Although this study analyses Swedish healthcare policy, the patterns could potentially be viewed in other national contexts and in other sectors. The influence of solutions in other countries, especially in the Nordic ones, can be viewed in the government documents. The evidence-informed approach spread from medicine to healthcare to adjacent areas, such as public health, social services, and education. This can be discerned also in the development of knowledge governance in Sweden. It is likely that many actors also in other countries have attempted to provide decision-makers with evidence or governing tools based on this notion, and contributed to an excess of directives, administration and treatments.

Highlighting policy dynamics emphasises the need for sound political processes rather than technical ones in decision making. Politics, for better or worse, plays a critical role in health policy and resource allocation. The challenge is how political processes can be improved. Comparing options and making trade-offs explicit is demanding, but a necessary form of political decision-making. Health systems need good political leaders than can make conflicting ideas, values and assumptions explicit when it comes to agreeing on the most urgent needs in resource allocation in healthcare. The importance of politics puts emphasis on skills such as systems thinking, as well as handling uncertainty and disagreement and channeling this positively. Resilience, perspective-taking, and engagement with the public are central. How political processes in resource allocation in healthcare can be improved is an area that needs more focus in research.

The focus of this study is on ideas and structures at a broader level than the narratives of distinct actors. However, this focus implies some limitations to the analysis; e.g. it does not say anything about different counter narratives and why some can become dominant narratives, or about inequalities in access to the “storytelling stage” and the silencing of particular narratives or other possible narratives. Although the contestation of ideas is evident in government documents, analysing the narratives of different actors, e.g. texts authored by groups outside of official government office, could offer more insight. It could provide more nuances about the meaning-making and power struggles in the development of healthcare policy and the evidence-informed approach.

## Conclusion

This study explores policy narratives underpinning evidence-informed decision-making within Swedish healthcare, tracing their formulation and development from 1992 to 2024. Four episodes with a dominant narrative in each emerged in the analysis.

The essence of these four policy narratives can be summarised as: 1) Information to decision-makers and deliberation between them will develop their knowledge and enable efficient and effective resource use in healthcare 2) Indicator-based quality monitoring and integration of data production and management will provide decision-makers with knowledge and enable nation-wide equality, equity and efficiency 3) Collaboration, synthesis and coordination will produce and apply best available knowledge as well as enable integrated and efficient governance and healthcare; and 4) Person-centeredness and emphasis on the local context for health professionals will enable a responsive, efficient and knowledge-based healthcare.

Together the four parallel narratives form a multi-faceted overall story of what goals the Swedish version of facilitating evidence-informed decision-making in healthcare is striving for, what problems it seeks to address, what solutions it offers, who has the knowledge, and how resources should be used. The findings offer insights into the governance of evidence-informed decision-making in healthcare. It contributes to a broader understanding of implications of the evidence-informed approach on healthcare policy, and how healthcare policy is shaped by competing ideas, values and assumptions. It also contributes to the theoretical debate on policy narratives, and discursive practice in general in healthcare governance.

## Data Availability

The documents used and analysed in the current study are available from the corresponding author on reasonable request.

## Abbreviations

EIDM: Evidence-informed decision-making
SBU: Swedish Council on Technology Assessment in Health Care (currently Swedish Agency for Health Technology Assessment and Assessment of Social Services)
NBHW: National Board of Health and Welfare
SALAR: Swedish Association of Local Authorities and Regions

## References

[1] World Health Organization. Evidence, policy, impact. WHO guide for evidence-informed decision-making. 1st ed. Geneva; 2022.

[2] Haby MM, Reveiz L, Thomas R, Jordan H. An integrated framework to guide evidence-informed public health policymaking: a framework for evidence-informed policy. J Public Health Pol. 2025;46(1):193–210. 10.1057/s41271-024-00535-9. 10.1057/s41271-024-00535-9 PMC11893451 39799247

[3] Clark EC, Burnett T, Blair R, Traynor RL, Hagerman L, Dobbins M. Strategies to implement evidence-informed decision making at the organizational level: a rapid systematic review. BMC Health Serv Res. 2024, Apr, 1;24(1):405. 10.1186/s12913-024-10841-3. 38561796 10.1186/s12913-024-10841-3 PMC10983660

[4] Kapiriri L. Does the narrative about the use of evidence in priority setting Vary across health Programs within the health sector: a case study of 6 Programs in a low-income national healthcare system. Int J Health Policy Manag. 2020, Jan, 21;1. 10.15171/ijhpm.2019.133. 10.15171/ijhpm.2019.133 PMC7719212 32610742

[5] Baltussen R, Jansen MP, Mikkelsen E, Tromp N, Hontelez J, Bijlmakers L, et al. Priority setting for universal health coverage: we need evidence-informed deliberative processes, not just more evidence on cost-effectiveness. Int J Health Policy Manag. 2016, June, 22;5(11):615–18. 10.15171/ijhpm.2016.83. 27801355 10.15171/ijhpm.2016.83 PMC5088720

[6] Jansen MP, Helderman JK, Boer B, Baltussen R. Fair processes for priority setting: putting theory into practice Comment on “Expanded HTA: enhancing Fairness and legitimacy”. Int J Health Policy Manag. 2016, July, 3;6(1):43–47. 10.15171/ijhpm.2016.85. 10.15171/ijhpm.2016.85 PMC5193505 28005541

[7] Cairney P. The politics of evidence-based policy making. In: Oxford research encyclopedia of politics. Oxford University Press; 2017.

[8] Cairney P, Oliver K. Evidence-based policymaking is not like evidence-based medicine, so how far should you go to bridge the divide between evidence and policy? Health Res Policy Sys. 2017, Dec;15(1):35, s12961-017-0192-x. 10.1186/s12961-017-0192-x. 10.1186/s12961-017-0192-x PMC5407004 28446185

[9] Parkhurst J. The politics of evidence (Open access): from evidence-based policy to the good governance of evidence. Oxford: Routledge; 2016. 196 p.

[10] Cairney P, Boaz A, Oliver K. Translating evidence into policy and practice: what do we know already, and what would further research look like? BMJ Qual Saf. 2023, May;32(5):251–53. 10.1136/bmjqs-2023-015911. 36948543 10.1136/bmjqs-2023-015911

[11] MacKillop E, Quarmby S, Downe J. Does knowledge brokering facilitate evidence-based policy? A review of existing knowledge and an agenda for future research. Policy Polit. 2020, Apr;48(2):335–53. 10.1332/030557319X15740848311069.

[12] Bergstedt E, Sandman L, Nedlund AC. Consolidating political leadership in healthcare: a mediating institution for priority-setting as a political strategy in a local health system. Health Econ Policy Law. 2024, July;19(3):337–52. 10.1017/S1744133124000021. 38449373 10.1017/S1744133124000021

[13] Head BW. Wicked problems in public policy: understanding and responding to complex challenges. Cham: Springer Nature; 2022. 176 p.

[14] Gough D, Boaz A. Applying the rational model of evidence-informed policy and practice in the real world. Evid Policy. 2017;13(1):3–5. 10.1332/174426417X14843185655356.

[15] Nedlund AC, Garpenby P. Puzzling about problems: the ambiguous search for an evidence-based strategy for handling influx of health technology. Policy Sci. 2014, Dec;47(4):367–86. 10.1007/s11077-014-9198-1.

[16] Strassheim H. A world of evidence: the global spread and silent politics of evidence cultures. Policy Soc. 2024;43(4):414–31. 10.1093/polsoc/puae029.

[17] Simons A, Schniedermann A. The neglected politics behind evidence-based policy: shedding light on instrument constituency dynamics. Policy Polit. 2021;49(4):513–29. 10.1332/030557321X16225469993170.

[18] MacKillop E, Downe J. Knowledge brokering organisations: a new way of governing evidence. Evid Policy. 2023;19(1):22–41. 10.1332/174426421X16445093010411.

[19] Onoda T. ‘Depoliticised’ regulators as a source of politicisation: rationing drugs in England and France. J Eur Public Policy. 2024, May, 3;31(5):1346–67. 10.1080/13501763.2023.2176909.

[20] Oliver K, Boaz A. Transforming evidence for policy and practice: creating space for new conversations. Palgrave Commun. 2019, May, 28;5(1):60. 10.1057/s41599-019-0266-1.

[21] Boaz A, Davies H, Fraser A, Nutley S, Eds.. What works Now? evidence-informed policy and practice. Bristol, UK: Policy Press; 2019.

[22] Cairney P, Oliver K, Wellstead A. To bridge the divide between evidence and policy: reduce ambiguity as much as uncertainty. Public Adm Rev. 2016, May;76(3):399–402. 10.1111/puar.12555.

[23] Jones MD, Anderson Crow D. How can we use the ‘science of stories’ to produce persuasive scientific stories? Palgrave Commun. 2017, Dec, 22;3(1):53. 10.1057/s41599-017-0047-7.

[24] Davidson B. Storytelling and evidence-based policy: lessons from the grey literature. Palgrave Commun. 2017, Sept, 12;3(1):17093. 10.1057/palcomms.2017.93.

[25] Mayer FW. Narrative politics : stories and collective action. 1st ed. New York: Oxford University Press; 2014. 193 p.

[26] Fischer F. Public policy as narrative: stories, frames, and metanarratives. In: Reframing public policy. 1st ed. Oxford: Oxford University Press; 2003. p. 161–80.

[27] Lowndes V. Narrative and storytelling. In: Stoker G, Evans M, editors. Evidence-based policy making in the social sciences. 1st ed. Bristol University Press; 2016. p. 103–22.

[28] Patterson M, Monroe KR. Narrative in political science. Annu Rev Polit Sci. 1998;1(1):315–31. 10.1146/annurev.polisci.1.1.315.

[29] Stone D. Policy paradox: the art of political decision making. 3rd ed. New York: W.W. Norton & Co; 2012. 408 p.

[30] Kay A. The dynamics of public policy: theory and evidence. Cheltenham: E. Elgar; 2006.

[31] Nedlund AC, Nordh J. Crafting citizen(ship) for people with dementia: how policy narratives at national level in Sweden informed politics of time from 1975 to 2013. J Educ Chang Aging Stud. 2015, Aug;34:123–33. 10.1016/j.jaging.2015.06.003. 10.1016/j.jaging.2015.06.003 26162732

[32] De Leeuw E, Clavier C, Breton E. Health policy – why research it and how: health political science. Health Res Policy Sys. 2014, Dec;12(1):55. 10.1186/1478-4505-12-55. 10.1186/1478-4505-12-55 PMC4246431 25248956

[33] Atkinson R. Narratives of policy: the construction of urban problems and urban policy in the official discourse of British government 1968–1998. Crit Soc Policy. 2000, May;20(2):211–32. 10.1177/026101830002000202.

[34] Greenhalgh T, Russell J. Evidence-based policymaking: a critique. Pbm. 2009, Mar;52(2):304–18. 10.1353/pbm.0.0085. 10.1353/pbm.0.0085 19395827

[35] Blumer H. Social problems as collective behavior. Soc Probl. 1971, Jan;18(3):298–306. 10.2307/799797.

[36] Boswell C, Geddes A, Scholten P. The role of narratives in migration policy-making: a research framework. Br J Polit Int Relat. 2011, Feb;13(1):1–11. 10.1111/j.1467-856X.2010.00435.x.

[37] Stone D. Causal stories and the formation of policy agendas. Polit Sci Q. 1989, June, 1;104(2):281–300. 10.2307/2151585.

[38] Roe E. Narrative policy analysis. Duke University Press; 1994.

[39] Wagenaar H. Meaning in action: interpretation and dialogue in policy analysis. London, New York: Routledge; 2015.

[40] Yanow D. Interpretation in policy analysis: on methods and practice. Crit Policy Stud. 2007, Mar, 1;1(1):110–22. 10.1080/19460171.2007.9518511.

[41] Miller HT. Policy narratives: the perlocutionary agents of political discourse. Crit Policy Stud. 2020;14(4):488–501. 10.1080/19460171.2020.1816483.

[42] Nedlund AC, Nordh J. Constructing citizens: a matter of labeling, imaging and underlying rationales in the case of people with dementia. Crit Policy Stud. 2018, July, 3;12(3):253–72. 10.1080/19460171.2017.1297730.

[43] Blomqvist P, Winblad U. Sweden. In: Immergut EM, Anderson KM, Devitt C, Popic T, editors. Health politics in Europe. 1st edn. Oxford: Oxford University Press; 2021. p. 164–204.

[44] Fredriksson M, Blomqvist P, Winblad U. Recentralizing healthcare through evidence-based guidelines - striving for national equity in Sweden. BMC Health Serv Res. 2014, Dec;14(1):509. 10.1186/s12913-014-0509-1. 25370710 10.1186/s12913-014-0509-1 PMC4226849

[45] Sandberg J, Persson B, Garpenby P. The dilemma of knowledge use in political decision-making: National guidelines in a Swedish priority-setting context. HEPL. 2019, Oct;14(4):425–42. 10.1017/S1744133118000233. 29986792 10.1017/S1744133118000233

[46] Socialdepartementet [Ministry of Health and Social Affairs]. Ds 2014:9. En samlad kunskapsstyrning för hälso- och sjukvård och socialtjänst [Ds 2014:9 A unified knowledge governance for health care and social services]. Stockholm: Fritze; 2014.

[47] Tavory I, Timmermans S. Abductive analysis: theorizing qualitative research. Chicago: The University of Chicago Press; 2014. 172 p.

[48] Ritchie J, Lewis J, editors. Qualitative research practice: a guide for social science students and researchers. London, Thousand Oaks, New Dehli: Sage Publications; 2003. 336 p.

[49] Socialdepartementet [Ministry of Health and Social Affairs]. Kommittédirektiv 1992:30 Hälso- och sjukvårdens finansiering och organisation [Committee Terms of Reference 1992:30 Financing and organisation of health care]. Stockholm: Regeringskansliet; 1992.

[50] Social departementet [Ministry of Health and Social Affairs]. SOU 1993:98 Hälso- och sjukvården i framtiden - tre modeller [SOU 1993:98 Healthcare in the future – three models]. Stockholm: Fritze; 1993.

[51] Finansdepartementet [Ministry of Finance]. Regeringens proposition 1993/94:100. Bilaga 6 till budgetpropositionen 1994. Socialdepartementet (femte huvudtiteln) [Government Bill 1993/94:100. Appendix 6 to the Budget Bill 1994. Ministry of Health and Welfare (fifth main title)]. Stockholm: Regeringskansliet; 1993.

[52] Socialdepartementet [Ministry of Health and Social Affairs]. Regeringens skrivelse 1996/97:66 Redogörelse för en överenskommelse om vissa ersättningar till sjukvårdshuvudmännen m.m. för år 1997 [Government communication 1996/97:66 Report on an agreement on certain compensations to health authorities etc. for 1997]. Stockholm: Regeringskansliet; 1996.

[53] Socialdepartementet [Ministry of Health and Social Affairs]. Kommittédirektiv 1997:135 Delegation för uppföljning av riktlinjer för prioriteringar inom hälso- och sjukvården [Committee Terms of Reference 1997:135 Delegation for follow-up of guidelines for priorities in healthcare]. Stockholm: Regeringskansliet; 1997.

[54] Socialdepartementet [Ministry of Health and Social Affairs]. SOU 1996:163 Behov och resuser i vården - en analys [SOU 1996:163 Needs and resources in healthcare – an analysis]. Stockholm: Fritze; 1996.

[55] Socialdepartementet [Ministry of Health and Social Affairs]. Regeringens skrivelse 2000/01:46. Redogörelse för en överenskommelse mellan staten och landstingen om ersättningar till hälso- och sjukvården för år 2001 [Government communication 2000/01:46. Report on an agreement between the state and the county councils on compensations to healthcare for the year 2001]. Stockholm: Regeringskansliet; 2000.

[56] Socialdepartementet [Ministry of Health and Social Affairs]. Regeringens skrivelse 2001/02:87. Redogörelse för en överenskommelse mellan staten och Landstingsförbundet om vissa ersättningar till hälso- och sjukvården för år 2002 [Government communication 2001/02:87. Report on an agreement between the state and the Association of county councils on compensations to healthcare for the year 2002]. Stockholm: Regeringskansliet; 2001.

[57] Socialdepartementet [Ministry of Health and Social Affairs]. SOU 2001:8 Prioriteringar i vården. Perspektiv för politiker, profession och medborgare [SOU 2001:8 Priorities in healthcare. Perspectives for politicians, professionals and citizens]. Stockholm: Fritze; 2001

[58] Socialdepartementet [Ministry of Health and Social Affairs]. SOU 1999:66. God vård på lika villkor? - om statens styrning av hälso- och sjukvården. [SOU 1999:66. Good care on equal terms? – on the state’s governance of health care]. Stockholm: Fritze; 1999.

[59] Finansdepartementet [Ministry of Finance]. Regeringens proposition 1994/95:100. Bilaga 6 till budgetpropositionen 1995. Socialdepartementet (femte huvudtiteln) [Government Bill 1994/95:100. Appendix 6 to the Budget Bill 1995. Ministry of Health and Welfare (fifth main title)]. Stockholm: Regeringskansliet; 1994.

[60] Socialdepartementet [Ministry of Health and Social Affairs]. Regeringens skrivelse 1997/98:51 Redogörelse för en överenskommelse om vissa ersättningar till sjukvårdshuvudmännen m.m. för år 1998 [Government communication 1997/98:51 Report on an agreement between the state and the county councils on certain compensations to healthcare for the year 1998]. Stockholm: Regeringskansliet; 1997.

[61] Socialdepartementet [Ministry of Health and Social Affairs]. Överenskommelse mellan staten och Landstingsförbundet om vissa ersättningar till hälso- och sjukvården för år 2005. Bilaga till protokoll nr 14 vid regeringssammanträde 2004-12-09 [Agreement between the State and the Association of County Councils on certain compensations to healthcare for 2005. Appendix to minutes no. 14 from the government meeting on 2004-12-09]. Stockholm: Regeringskansliet; 2004.

[62] Socialdepartementet [Ministry of Health and Social Affairs]. Dagmaröverenskommelse 2007 – överenskommelse mellan staten och Landstingsförbundet om vissa ersättningar till hälso- och sjukvården [Dagmar Agreement 2007 – agreement between the state and the Association of Swedish County Councils on certain compensations to healthcare]. Stockholm: Regeringskansliet; 2006.

[63] Socialdepartementet [Ministry of Health and Social Affairs]. Dagmaröverenskommelse 2008 - överenskommelse mellan staten och Sveriges Kommuner och Landsting om vissa ersättningar till hälso- och sjukvården [Dagmar Agreement 2008 – agreement between the state and the Swedish Association of Local Authorities and Regions on certain compensations to healthcare]. Stockholm: Regeringskansliet; 2007.

[64] Finansdepartementet [Ministry of Finance]. Regeringens proposition 2012/13:1. Budgetproposition 2013 Utgiftsområde 9: Hälsovård, sjukvård och social omsorg [Government Bill 2012/13:1. Budget Bill 2013. Expenditure area 9: Health care, medical care and social welfare]. Stockholm: Regeringskansliet; 2012.

[65] Finansdepartementet [Ministry of Finance]. Regeringens proposition 2010/11:1. Budgetproposition 2011 Utgiftsområde 9: Hälsovård, sjukvård och social omsorg [Government Bill 2010/11:1. Budget Bill 2011. Expenditure area 9: Health care, medical care and social welfare]. Stockholm: Regeringskansliet; 2010.

[66] Finansdepartementet [Ministry of Finance]. Regeringens proposition 2011/12:1. Budgetproposition 2012 Utgiftsområde 9: Hälsovård, sjukvård och social omsorg [Government Bill 2011/12:1. Budget Bill 2012. Expenditure area 9: Health care, medical care and social welfare]. Stockholm: Regeringskansliet; 2011.

[67] Socialdepartementet [Ministry of Health and Social Affairs]. SOU 2007:10 Hållbar samhällsorganisation med utvecklingskraft [SOU 2007:10 Sustainable societal organisation with development power]. Stockholm: Fritze; 2007.

[68] Socialdepartementet [Ministry of Health and Social Affairs]. SOU 2009:11 En nationell cancerstrategi för framtiden [SOU 2009:11 A national cancer strategy for the future]. Stockholm: Fritze; 2009.

[69] Socialdepartementet [Ministry of Health and Social Affairs]. SOU 2011:65 Statens roll i framtidens vård- och omsorgssystem – en kartläggning. [SOU 2011:65 The role of the state in the healthcare and social care system of the future – a survey]. Stockholm: Fritze; 2011.

[70] Socialdepartementet [Ministry of Health and Social Affairs]. SOU 2012:33 Gör det enklare! Slutbetänkande av Statens vård- och omsorgsutredning [SOU 2012:33 Make it easier! Final report by the Government Inquiry on Health and Social Care]. Stockholm: Fritze; 2012.

[71] Socialdepartementet [Ministry of Health and Social Affairs]. Kommittédirektiv 2013:104. En nationell samordnare för effektivare resursutnyttjande inom hälso- och sjukvården [Committee Terms of Reference 2013:104. A national coordinator for more efficient use of resources in health care]. Stockholm: Regeringskansliet; 2013

[72] Socialdepartementet [Ministry of Health and Social Affairs]. SOU 2016:2 Effektiv vård [SOU 2016:2 Effective health care]. Stockholm: Wolters Kluwer; 2016.

[73] Finansdepartementet [Ministry of Finance]. Regeringens proposition 2015/16:1. Budgetproposition 2016 Utgiftsområde 9: Hälsovård, sjukvård och social omsorg [Government Bill 2015/16:1. Budget Bill 2016. Expenditure area 9: Health care, medical care and social welfare]. Stockholm: Regeringskansliet; 2015.

[74] Socialdepartementet [Ministry of Health and Social Affairs]. Standardiserade vårdförlopp - Jämlik och effektiv vård med god kvalitet. Överenskommelse mellan staten och Sveriges Kommuner och Landsting 2019. Bilaga till protokoll vid regeringssammanträde 2019-05-16 nr 1:23 [Standardised care pathways – equal and efficient care with good quality. Agreement between the state and the Swedish Association of Local Authorities and Regions 2019. Appendix to minutes from government meeting 2019-05-16 no 1:23]. Stockholm: Regeringskansliet; 2019.

[75] Finansdepartementet [Ministry of Finance]. Regeringens proposition 2016/17:1. Budgetproposition 2017 Utgiftsområde 9: Hälsovård, sjukvård och social omsorg [Government Bill 2016/17:1. Budget Bill 2017. Expenditure area 9: Health care, medical care and social welfare]. Stockholm: Regeringskansliet; 2016.

[76] Socialdepartementet [Ministry of Health and Social Affairs]. Patientsäkerhet, nationella kvalitetsregister m.m. 2020 – överenskommelse mellan staten och Sveriges Kommuner och Regioner. Bilaga till protokoll vid regeringssammanträde 2019-12-19 nr 1:28 [Patient safety, national quality registers etc. 2020 – agreement between the state and the Swedish Association of Local Authorities and Regions. Appendix to minutes from government meeting 2019-12-19 no 1:28]. Stockholm: Regeringskansliet; 2019.

[77] Socialdepartementet [Ministry of Health and Social Affairs]. Sammanhållen, jämlik och säker vård 2023 – överenskommelse mellan staten och Sveriges Kommuner och Regioner. Bilaga till regeringsbeslut 2023‑01‑26 nr II:4 [Coordinated, equal and safe care 2023 – agreement between the state and the Swedish Association of Local Authorities and Regions. Appendix to government decision 2023‑01‑26 no. II:4]. Stockholm: Regeringskansliet; 2023.

[78] Finansdepartementet [Ministry of Finance]. Regeringens proposition 2022/23:1. Budgetproposition 2023 Utgiftsområde 9: Hälsovård, sjukvård och social omsorg [Government Bill 2022/23:1. Budget Bill 2023. Expenditure area 9: Health care, medical care and social welfare]. Stockholm: Regeringskansliet; 2022.

[79] Finansdepartementet [Ministry of Finance]. Regeringens proposition 2020/21:1. Budgetproposition 2021 Utgiftsområde 9: Hälsovård, sjukvård och social omsorg [Government Bill 2020/21:1. Budget Bill 2021. Expenditure area 9: Health care, medical care and social welfare]. Stockholm: Regeringskansliet; 2020.

[80] Socialdepartementet [Ministry of Health and Social Affairs]. SOU 2020:36. Ett nationellt sammanhållet system för kunskapsbaserad vård – ett system, många möjligheter [SOU 2020:36 A national coherent system for knowledge‑based health care – one system, many possibilities]. Stockholm: Norstedts Juridik; 2020.

[81] Socialdepartementet [Ministry of Health and Social Affairs]. Gemensam inriktning för en sammanhållen och ändamålsenlig kunskapsstyrning för hälso‑ och sjukvården. Bilaga till beslut vid regeringssammanträde 2024‑03‑21 nr II:1 [Joint direction for a coherent and appropriate knowledge governance for health care. Annex to decision at government meeting 2024‑03‑21 no II:1]. Stockholm: Regeringskansliet; 2024.

[82] Loughlin M, Buetow S, Cournoyea M, Copeland SM, Chin-Yee B, Fulford KWM. Interactions between persons—knowledge, decision making, and the co-production of practice. Evaluation Clin Pract. 2019, Dec;25(6):911–20. 10.1111/jep.13297. 10.1111/jep.13297 31733025

